# Computed Tomography and Magnetic Resonance Imaging Are Equivalent in Mensuration and Similarly Inaccurate in Grade and Type Predictability of Canine Intracranial Gliomas

**DOI:** 10.3389/fvets.2017.00157

**Published:** 2017-09-25

**Authors:** Krystina L. Stadler, Jeffrey D. Ruth, Theresa E. Pancotto, Stephen R. Werre, John H. Rossmeisl

**Affiliations:** ^1^Department of Small Animal Clinical Sciences, VA-MD College of Veterinary Medicine, Blacksburg, VA, United States

**Keywords:** brain tumor, computed tomography, magnetic resonance imaging, canine, glioma, neuroimaging, neurooncology

## Abstract

While magnetic resonance imaging (MRI) is the gold-standard imaging modality for diagnosis of intracranial neoplasia, computed tomography (CT) remains commonly used for diagnosis and therapeutic planning in veterinary medicine. Despite the routine use of both imaging modalities, comparison of CT and MRI has not been described in the canine patient. A retrospective study was performed to evaluate CT and MRI studies of 15 dogs with histologically confirmed glioma. Multiple lesion measurements were obtained, including two-dimensional and volumetric dimensions in pre-contrast and post-contrast images. Similar measurement techniques were compared between CT and MRI. The glioma type (astrocytoma or oligodendroglioma) and grade (high or low) were predicted on CT and MRI independently. With the exception of the comparison between CT pre-contrast volume to T2-weighted MRI volume, no other statistical differences between CT and MRI measurements were identified. Overall accuracy for tumor grade (high or low) was 46.7 and 53.3% for CT and MRI, respectively. For predicted tumor type, accuracy of CT was 53.3% and MRI and MRI 60%. Based on the results of this study, both CT and MRI contrast measurement techniques are considered equivalent options for lesion mensuration. Given the low-to-moderate predictability of CT and MRI in glioma diagnosis, histopathology remains necessary for accurate diagnosis of canine brain tumors.

## Introduction

Magnetic resonance imaging (MRI) is the gold-standard imaging modality for diagnosis of intracranial neoplasia. Prior to MRI, computed tomography (CT) was considered the gold-standard imaging modality for intra-axial lesions. Following the adoption of MRI as the diagnostic modality of choice in human and veterinary medicine for intracranial disease, the comparison of diagnostic findings and predictability between CT and MRI has been minimally explored in the literature. To the authors’ knowledge, only one study compares CT and MRI in diagnosing intra-axial gliomas (1). This study compared CT to MRI in its accuracy to detect histological tumor margins in experimentally induced gliomas in a canine model. It found that MRI was superior to CT in tumor margin detection and MRI with gadolinium contrast superior to non-contrast MRI and CT. The remaining literature available in human medicine compares CT to MRI in diagnosis of intracranial metastases, midline tumors, and meningiomas (2–4). Conclusions within these studies vary, with one concluding neither CT nor MRI was superior and the others finding MRI to be superior.

In veterinary medicine, MRI is the imaging modality of choice for diagnosis of intracranial lesions. CT, however, is more widely available than MRI in and is often used for radiation therapy planning for treatment of solitary intra-axial neoplasia (5) or stereotactic biopsy (6, 7) and remains the diagnostic modality of choice when MRI is not available. In addition, CT is often preferred for imaging of the extra-calvarial lesions of the head, in which concurrent intra-axial lesions may be detected. Given the continued use of CT as a sole or adjunct imaging modality in patients with brain tumors, the specific aims of the study were to compare lesion mensuration and predictability of tumor type and grade between CT and MRI in canine patients with histologically confirmed gliomas.

## Materials and Methods

Retrospective review of the Virginia-Maryland Veterinary Teaching Hospital’s (VA-MD VTH) Picture Archiving and Communications system (PACS) and medical records were performed for client-owned canine patients with a histopathologically confirmed solitary intra-axial glioma and an MRI and CT of the brain performed within 1 month of each other. Histopathological diagnosis was performed according to World Health Organization (WHO) criteria (8, 9) by anatomic pathologists with a focus on neuropathology. Patients were not included in if the imaging studies did not include contrast administration. If beam-hardening artifact was present on the CT images at the area of interest due to the presence of stereotactic or therapeutic device, the patient was not included in the study. As convention at our institution for post-contrast CT images, patients received iopromide (370 mgI/ml, Ultravist^®^) at a dose of 0.45 ml/kg (814 mgI/kg). For post-contrast MRI performed at VA-MD VTH, all patients received gadopentetate dimeglumine (0.5 mmol/ml, Magnevist^®^) at a dose of 0.2 ml/kg.

All images were anonymized for blinded image review using a 4-digit number generated by a random number generator (http://random.org). The CT and MRI study for the same patient had a different randomized number. CT images were reviewed first, and at least 2 weeks apart from MRI images. The thinnest slice CT pre- and post-contrast and T1-weighted (T1W) pre- and post-contrast and T2-weighted (T2W) images, all transverse, were available for review. All images were reviewed on an external workstation (OsiriX v8.5.1). The images were reviewed independently by three reviewers; two radiologists (Jeffrey D. Ruth and Krystina L. Stadler) and one neurologist (Theresa E. Pancotto). The reviewers were aware that all patients had histological confirmation of glioma but were blinded to type and grade.

Based on diagnostic imaging findings previously published for predicting grade and glial tumor type in veterinary medicine (10–12), reviewers predicted the grade (high or low) and glial tumor type (astrocytoma vs. oligodendroglioma) of each lesion on CT and MRI images independent of the imaging modality. Accuracy of predicting the lesion grade and type on CT and MRI compared to histopathological diagnosis was performed using the majority agreement based on the three reviewers (Krystina L. Stadler, Theresa E. Pancotto, and Jeffrey D. Ruth, ≥2 out of 3 agreed). In addition, agreement between CT and MRI tumor grade and type independent of histopathology diagnosis was assessed.

**Figure 1:**
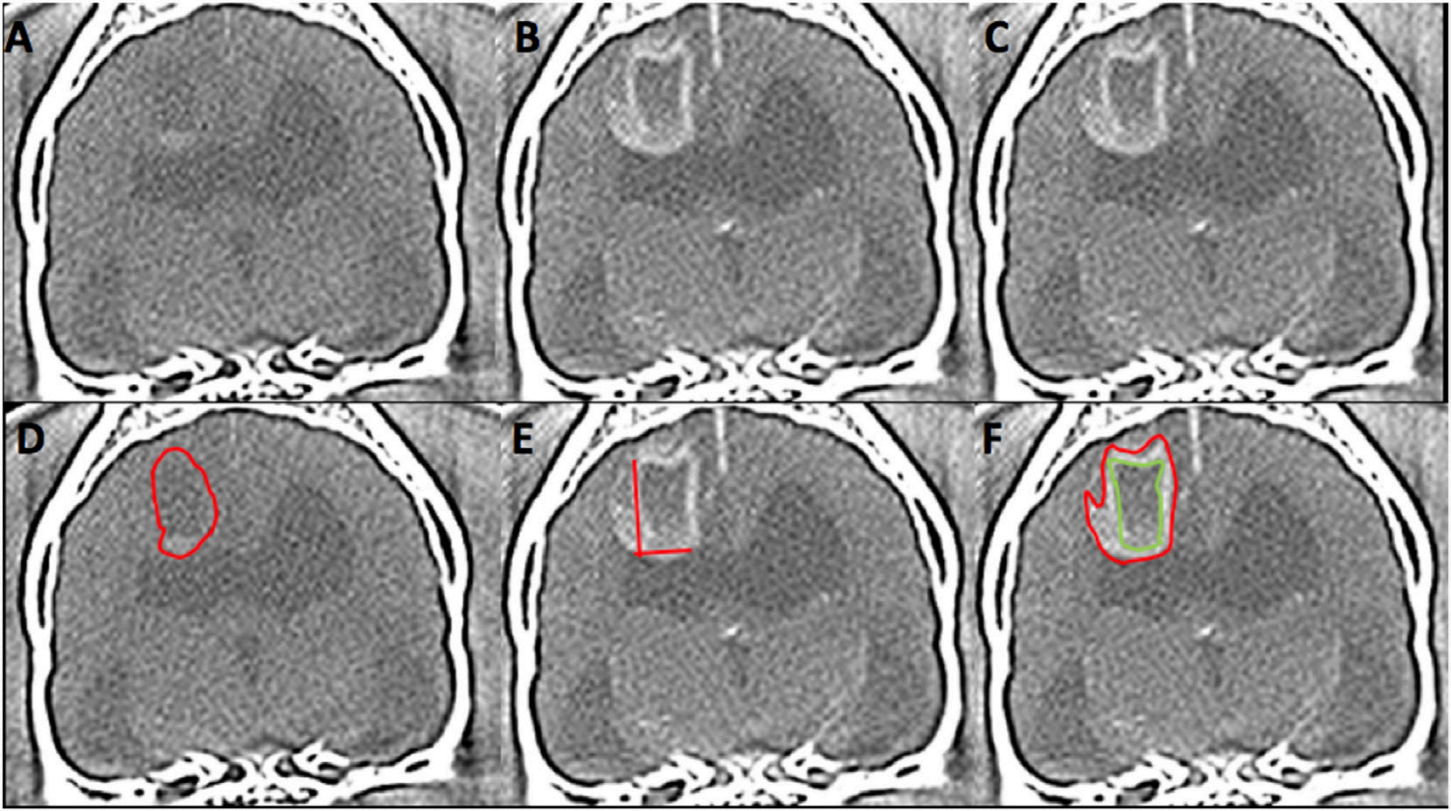
Computed tomography (CT) images from 6 year-old female spayed French bulldog with histological high-grade astrocytoma in the dorsal cerebrum at the level of the interthalamic adhesion. This lesion was diagnosed on CT as high grade (3/3 reviewers) and an oligodendroglioma (2/3). Panels **(A,D)** represent pre-contrast images. The red outline on image **(D)** represents the hand-drawn region of interest (ROI) for volume calculation. Panels **(B,C,E,F)** are post-contrast images. The two lines on image **(E)** represent the two-dimensional McDonald method of lesion measurement. The red outline on image **(F)** represents the hand-drawn ROI for total lesion volume. The green outline represents non-contrast enhancing (CE) lesion volume and the space between the two outlines represents the ROI for CE lesion only. (Images displayed with a window width: 350, Window level: 40.)

**Figure 2:**
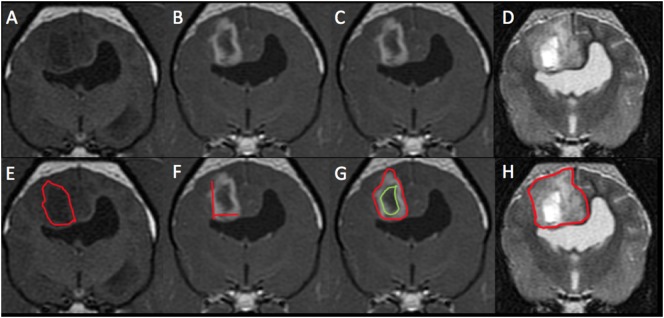
Magnetic resonance imaging (MRI) images from the same patient from Figure 1 with histological high-grade astrocytoma. This mass was diagnosed on MRI as high grade (3/3 reviewers) and an astrocytoma (2/3). Panels **(A,E)** represent pre-contrast T1-weighted (T1W) images. The red outline on image **(E)** represents the hand-drawn region of interest (ROI) for volume calculation. Panels **(B,C,F,G)** are T1W post-contrast images. The two lines on image **(F)** represent the two-dimensional McDonald method of lesion measurement. The red outline on image **(G)** represents the hand-drawn ROI for total lesion volume. The green outline represents non-contrast enhancing (CE) lesion volume and the space between the two outlines represents the ROI for CE lesion only. Panels **(D,H)** are T2-weighted pre-contrast images. The red outline on image **(H)** represents T2 hyperintense volume.

The mensurations performed on CT and MRI on the intra-axial lesions are detailed in Table 1 and Figures 1 and 2. The two-dimensional (2D) contrast enhancement measurement was obtained using the post-contrast CT and MRI transverse images by the 2D Macdonald method (13–15), involving continuous length × height dimension of contrast enhancement at the level of the greatest tumor contrast enhancement and diameter. For volumetric measurements, a free drawn region of interest was drawn on each slice surrounding the lesion and compute volume tool on OsiriX was used. T2 and T1 post-contrast volumetric measurements were performed similar to previous described methods (14–20). Volumetric measurements of T2 images included all continuous T2 hyperintensity surrounding the lesion (peritumoral edema). T1 pre-contrast volumetric measurements did not include perilesion T1 hypointesity (peritumoral edema). A zero measurement was assigned if no measurement could be performed either because the lesion was not identified or the desired measurement could not be obtained. Viewer measurements were averaged between the observers and statistical analysis of this data performed.

**Table 1:** Summary of measurements performed on available imaging sequences.

Computed tomography (CT) pre-contrast	CT post-contrast	T1-weighted (T1W) transverse pre-contrast	T1W post-contrast	T2-weighted (T2W)
	Two-dimensional (2D) contrast dimensions		2D contrast dimensions	
Total lesion volume (cm^3^)	Total lesion volume (cm^3^)	Total lesion volume (cm^3^)	Total lesion volume (cm^3^)	Total T2W hyperintense volume (cm^3^)
	Volume of contrast enhancing (CE) region only (cm^3^)		Volume of CE region only (cm^3^)	
	Volume of non-CE region only (cm^3^)		Volume of non-CE region only (cm^3^)	

For statistical analysis of acquired measurements, CT pre-contrast volume was compared to CT post-contrast volume, T1W pre-contrast volume and T2W pre-contrast volume independently. CT 2D contrast dimensions were compared to MRI 2D contrast dimensions. CT post-contrast measurements of contrast enhancing (CE) total volume, CE portion only volume, and non-CE volume were compared independently with their respective MRI T1W post-contrast measurements. Normal probability plots showed that CT contrast-enhanced total volume compared to T1W post-contrast total volume, CT pre-contrast total volume compared to T2W post total volume, CT CE volume only compared to MRI CE volume only measurements were skewed. Accordingly, these were summarized as medians. CT pre-contrast volume compared to T1W pre-contrast volume and compared to T2W volume measurements followed a normal distribution and were summarized as least squares means (SE). Comparisons of interest between measurements were made using the Wilcoxon signed rank test (CT no contrast volume to CT with contrast volume, CT 2D to MRI 2D, CT contrast volume to T1W plus contrast volume, CT pre-contrast volume to T1W pre-contrast volume) and mixed model analysis of variance followed by Tukey’s procedure for multiple comparisons (CT pre-contrast volume to T1W pre-contrast volume to T2W pre-contrast volume). Statistical significance was set at *p* < 0.05. All analyses were performed using SAS version 9.4 (Cary, NC, USA).

## Results

A total of 15 dogs fit the inclusion criteria. Breeds included mixed breed dog (*n* = 4), Boston terrier (3), Boxer (2), miniature Schnauzer, Golden retriever, Dachshund, English bulldog, French bulldog, and Labrador retriever (1 each). The average patient age was 8.3 years (range: 6–13 years). Ten were spayed females and five were castrated males. The average patient weight was 21.6 kg (range: 7.6–44.2 kg).

One of the 15 dogs did not have a pre-contrast CT; this dog was excluded from comparisons involving pre-contrast CT images. All CT examinations were performed at VA-MD veterinary teaching hospital using the in-house 16-slice CT scanner (Toshiba Aquillon 16, Japan). The location of the MRI scan varied with 5 patients having the MRI performed in-hospital (1.5 T Phillips Intera, USA), 10 performed at various referral institutions (1 with a 0.2-T MRI, 1 with a 1-T MRI, and 8 with a 1.5-T MRI). All CT images were evaluated at 1-mm slice thickness. MRI slice thickness varied with a range from 3.0 to 5.0 mm.

Histopathological diagnosis was achieved by stereotactic biopsy in 11 dogs and necropsy in 4 dogs. On histopathology, 10 gliomas were identified as high grade (WHO grade III or IV) and 5 were identified as low grade (WHO grade II). Eleven astrocytomas and four oligodendrogliomas were identified; of these eight astrocytomas and two oligodendrogliomas were high grade and three astrocytomas and two oligodendrogliomas were low grade. On imaging, all three observers agreed on the same grade in 9/15 (60%) of patients for CT and 8/15 (53.3%) patients for MRI. Comparing predicted tumor grade (either by 2/3 observer agreement or when all observers agreed), CT had an overall accuracy of 46.7% (7/15) and MRI had an overall accuracy of 53.3% (8/15). When comparing CT predicted grade to MRI predicted grade, agreement was seen in 9/15 cases (60%). In cases where CT and MRI diagnosis agreed, accuracy between histopathological grades was 44.4% (4/9). Sensitivity, specificity, positive predictive value (PPV), and negative predictive value (NPV) for high and low grade for both CT and MRI are detailed in Tables 2 and 3, respectively. Contrast enhancement volume only between CT and MRI and histopathological grade is detailed in Table 4.

**Table 2:** Sensitivity, specificity, positive predictive value (PPV), and negative predictive value (NPV) for computed tomography (CT) glioma grading.

CT grade	Sensitivity (%)	Specificity (%)	PPV (%)	NPV (%)
High	60	20	60	20
Low	20	60	25	60

**Table 3:** Sensitivity, specificity, positive predictive value (PPV), and negative predictive value (NPV) for magnetic resonance imaging (MRI) glioma grading.

MRI grade	Sensitivity (%)	Specificity (%)	PPV (%)	NPV (%)
High	60	40	67	33
Low	33	33	40	60

**Table 4:** Contrast enhancing (CE) tumor volume for high- and low-grade gliomas on computed tomography (CT) and magnetic resonance imaging (MRI).

Tumor grade	CT-average CE volume cm^3^ (range)	CT SD	MRI-average CE volume cm^3^ (range)	MRI-SD
Low	1.13 (0.15–2.79)	0.35	1.44 (0.2–3.22)	1.5
High	2.4 (0–5.27)	2.49	2.13 (0.8–3.08)	2.05

All tumors were identified as intra-axial, except for one which was identified by all three reviewers as extra-axial and was histologically confirmed to be an optic chiasm astrocytoma. For type of glioma, all three observers agreed in 6/15 (40%) patients for CT and 4/15 (26.7%) patients for MRI. Using predicted tumor type, either majority or unanimous observer agreement, CT had an accuracy of 53.3% (8/15) and MRI an accuracy of 60% (9/15). When comparing CT predicted tumor type to MRI predicted tumor type, agreement was seen in 11/15 cases (73.3%). In cases where CT and MRI tumor type agreed, accuracy (sensitivity) between histopathology was 54.5% (6/11), specificity for this group was also 54.5%. Sensitivity, specificity, PPV, and NPV for glioma type for both CT and MRI are detailed in Tables 5 and 6, respectively. For these analyses, the MRI diagnosed extra-axial tumor was removed from the study population. Since no tumors were misclassified as astrocytomas on CT and diagnosed as oligodendrogliomas on histopathology, the CT specificity and PPV for astrocytoma and sensitivity and NPV for oligodendroglioma could not be calculated.

**Table 5:** Sensitivity, specificity, positive predictive value (PPV), and negative predictive value (NPV) for computed tomography (CT) glioma type prediction.

CT diagnosis	Sensitivity (%)	Specificity (%)	PPV (%)	NPV (%)
Oligodendroglioma	NA	45.5	33.3	NA
Astrocytoma	45.5	NA	NA	33.3

**Table 6:** Sensitivity, specificity, positive predictive value (PPV), and negative predictive value (NPV) for magnetic resonance imaging (MRI) glioma type prediction.

MRI diagnosis	Sensitivity (%)	Specificity (%)	PPV (%)	NPV (%)
Oligodendroglioma	66.7	36.4	33.3	87.5
Astrocytoma	63.6	66.7	87.5	66.7

The averages, median, range, SD, and variation coefficient for CT and MRI measurements are detailed in Tables 7 and 8, respectively. On CT, at least one reviewer could not identify the lesion for mensuration pre- or post-contrast in the same two cases and therefore a 0 was assigned. On MRI, the lesion could not be identified on T1 pre-contrast images on two patients, both of which had lesions identified and measured on post-contrast images. Four masses were uniformly CE and thus a measurement of a non-CE volume could not be performed. In one patient with a small (0.4 cm^3^ T1 + C lesion), one reviewer was unable to measure two dimensions and therefore recorded 0 and the other two had a small 2D measurement (0.5 cm^3^). The comparisons acquired, the statistical test used, the statistical test SD, and *p*-value are highlighted in Table 9. The only statistically significant comparison was the CT pre-contrast volume compared to T2W volume.

**Table 7:** Summary of median measurement, range, SD, and variation coefficient for computed tomography (CT) measurements.

CT measurement	Median	Range	SD	Variation coefficient
Two-dimensional (2D) (cm^2^)	1.62	0–6	0.79	0.63
CT pre-contrast volume (cm^3^)	2.3	0–9.88	1.9	1.68
CT post-contrast volume (cm^3^)	2.05	0–10.18	1	0.53
CT post-contrast contrast enhancing (CE) volume (cm^3^) only	1.15	0–7.18	0.64	0.37
CT post-contrast non-CE volume only	0.53	0–6.5	0.43	0.5

**Table 8:** Summary of median measurement, range, and SD for magnetic resonance imaging (MRI) measurements.

MRI measurement	Median	Range	SD	Variation coefficient
Two-dimensional (cm^2^)	1.61	0–7.2	1.03	0.58
MRI T1-weighted (T1W) pre-contrast volume (cm^3^)	3.22	0–11.3	0.3	0.3
MRI T2-weighted contrast volume (cm^3^)	5.13	0.07–13.41	0.81	0.19
MRI T1W post-contrast volume (cm^3^)	2.6	0.19–10.03	0.87	0.32
MRI T1W post-contrast contrast enhancing (CE) volume only (cm^3^)	1.36	0–9.28	0.85	0.48
MRI T1W post-contrast non-CE volume only	1.02	0–4.05	0.42	0.5

**Table 9:** Computed tomography (CT) to magnetic resonance imaging (MRI) comparisons, statistical test used, and *p*-value.

Comparison	Statistical test	*p*-Value
CT pre-contrast volume to CT post-contrast volume	Wilcoxon signed rank	0.86
Post-contrast: two-dimensional CT to T1-weighted (T1W) MRI	Wilcoxon signed rank	0.65
Post-contrast: CT to T1W MR contrast enhancing (CE) volume only	Wilcoxon signed rank	0.56
Post-contrast: CT to T1W non-CE volume only	Wilcoxon signed rank	0.1970
CT pre-contrast volume to T1W pre-contrast volume	Mixed model ANOVA	0.41
CT pre-contrast volume to T2-weighted (T2W) volume	Mixed model ANOVA	**0.03**
T1W pre-contrast volume to T2W volume	Mixed model ANOVA	0.35

*The bold font indicates statistical significance*.

## Discussion

To the author’s knowledge, this is the first report detailing the application of previously described imaging characteristics of canine glioma to predict tumor types and grades. Compared to human medicine, our ability to predict glioma grade in dogs based on imaging characteristics was low. In human medicine, reports have discussed predictability of tumor grade on conventional MRI images with accuracy widely varying between studies from 65 to 94% (21–23). Law et al. (22) looked at the sensitivity, specificity, PPV, and NPV for predicting high-grade gliomas with conventional MRI and found it to be 72.5, 65, 86.1, and 44.1%, respectively. These values are greater than found with our data in dogs with high-grade gliomas on both CT and MRI. An additional human study (24) found that for low-grade astrocytomas, a 50% false positive rate was observed on MRI in predicting grade. This finding is similar to our data for high-grade gliomas, and less than what this study found with low-grade gliomas for both MRI and CT. One possible reason is the observer subjectivity with respect to the degree of contrast enhancement as it correlates to high-grade gliomas. However, subjective criteria detailed in previous veterinary literature were used for this study (12). Although objective methods of contrast enhancement quantification have been described in humans, they have not been evaluated in veterinary medicine (19). One study in dogs reported the presence of contrast enhancement as being significantly associated with high-grade compared to low-grade gliomas (10). An additional study found the absence of or mild contrast enhancement significantly correlated low-grade gliomas (11). In our study, only one tumor failed to have any contrast enhancement on CT and was diagnosed unanimously as a low-grade tumor both on CT and MRI. Interestingly, this lesion was a high-grade glioma on histopathology. When evaluating quantitative CE volumes between low- and high-grade tumors (Table 3), a large overlap exists between both low- and high-grade gliomas. This finding supports those of the previously reported veterinary literature (10, 11) and suggests that subjective degree and volume mensuration of contrast enhancement may not correlate with histopathological grade.

Most literature in human medicine regarding imaging predictability of gliomas is focused on glioma grade and not type. One report found that conventional MRI sequences were able to correctly differentiate 80.6–83.3% of grade II–IV astrocytomas (21). Another study, comparing primary intra-axial tumors had an accuracy of 94% (23). Results of these studies are both greater than the overall accuracy reported here using CT, MRI, or both modalities. As mentioned in previous veterinary reports (10, 11), overlap and redundancy of findings are noted between tumor types and likely the cause of low-to-moderate tumor type predictability in our study. Potentially also impacting our predictability for grade and glioma type was using a majority agreement between reviewers, in comparison to a solitary reviewer to predict diagnosis. This type of agreement is similar to what occurs in our hospital on clinical cases.

To the authors’ knowledge, comparison of mensuration of gliomas using CT and MRI has not been previously described in dogs. Both contrast-enhanced 2D measurements and three-dimensional (3D) volume measurements are described in measurement techniques for clinical trial monitoring of intra-axial tumors (14). In human literature, comparison of 2D to 3D volumetric measurements in terms of therapeutic response has been found to perform similarly (20, 25). No current consensus on glioma mensuration in veterinary medicine exists; however, using the same measurement technique between serial imaging for evaluating response to therapy is recommended (15). Based on the data from this study, no statistical significance exists between CT and MRI when comparing similar measurement techniques, with the exception of comparing CT pre-contrast volume to T2W volume. This is likely due to the inclusion of perilesional edema in the T2W measurements. For better comparison of CT post-contrast and T1W post-contrast to the pre-contrast images, perilesional edema was not included in the measurement. However, it is likely that a small amount of perilesional edema was included within the pre-contrast volumetric measurement given the delineation between edema and mass was not consistently well defined. Despite the lack of perilesional edema inclusion within the T1W pre-contrast images, no statistical difference was appreciated between T1 pre-contrast and T2W volumetric measurements. To the authors’ knowledge, the volumetric measurement of CT and T1W pre-contrast images has not been described. Interestingly, in multiple cases on both CT and MRI, at least one reviewer was unable to complete the measurement, either due to lack of detection, mass shape, or lack of heterogeneity (in the cause of CE vs. non-CE volume). On CT, two gliomas were not visualized on post-contrast images by at least one reviewer; however, all reviewers were able to visualize and measure the glioma volume of the glioma on T1W post-contrast images. Although not statistically significant, this finding may argue for MRI a preferred imaging modality. In addition, since the statistical findings support that CT and MRI may be used interchangeably to measure gliomas; however, from a clinical standpoint, maintaining the same imaging modality to evaluate for therapeutic response, should be performed, if possible (15).

Multiple limitations of this study exist. Since this study is retrospective in nature, the timeframe between CT and MRI could not be closely controlled, however, was specified in the inclusion criteria. Given that no significant differences in the majority of the mensuration parameters existed between the two modalities, the specified timeframe between studies likely did not affect the outcome. The majority of this study’s histopathological diagnoses were achieved *via* stereotactic biopsy. Variable reliability of histological grading of stereotactic biopsy has been reported in human medicine (26, 27). Thus, it may be possible that some of the gliomas included in this study were erroneously graded and therefore contributing to the lower predictability of this study in comparison to human studies that used excisional biopsies for histopathology. An additional limitation is the relatively small sample size included in this study, largely due to overall disease prevalence, and requirements for both histopathological tumor confirmation and performance of contemporaneous CT and MRI imagingstudies.

In conclusion, the results of this study suggest that both conventional CT and MRI have a low-to-moderate ability to predict types and grades of canine gliomas, and that histological evaluation is necessary for accurate diagnosis of canine brain tumors. Based on the results of this study for conventionally used contrast-enhanced measurement techniques, CT and MRI have no significant difference and thus both are considered reasonable options for tumor mensuration. Further studies are required to determine if the discriminatory abilities of CT and MRI are improved with the addition of techniques such as dynamic contrast-enhanced imaging, diffusion-weighted imaging, or MR spectroscopy and to assess if significant difference exists between the modalities for assessment of therapeutic response.

## Ethics Statement

This study is retrospective in nature and not all imaging (MRI) was performed at VA-MD Veterinary Teaching Hospital. All patient imaging performed at VA-MD VTH was performed in accordance to Virginia Tech IACUC 14-235CVM and 15-122CVM. The obtaining and use of imaging studies performed outside VA-MD VTH was approved by written client consent.

## Author Contributions

KS: primary researcher, responsible for study development and organization, including the acquisition of patients, anonymizing and distributing cases, imaging and patient data and analyzing and organizing reviewer data. One of three image reviewers. Drafting and editing of manuscript. JRuth: assist in drafting study design. One of three image reviewers. Internal manuscript revision. TP: assist in drafting study design. One of three image reviewers. Internal manuscript revision. SW: statistician, responsible for performing data statistics and drafting the manuscript portion pertaining to. Internal manuscript revision. JRossmeisl: responsible for initial prospective clinical trial in which patients were retrospectively pulled from and assisted in getting all images needed for this study. Assist in drafting study design. Internal manuscript revision.

## Conflict of Interest Statement

The authors declare that the research was conducted in the absence of any commercial or financial relationships that could be construed as a potential conflict of interest.

## Abbreviations

CT, computed tomography; MRI, magnetic resonance imaging; 2D, two-dimensional; 3D, three-dimensional; PACS, picture archiving and communication system; T1W, T1-weighted; T2W, T2-weighted; VA-MD VTH, Virginia-Maryland Veterinary Teaching Hospital; WHO, World Health Organization.
